# Assessment of Protocol Impact on Subjectivity Uncertainty When Analyzing Peripheral Blood Mononuclear Cell Flow Cytometry Data Files

**DOI:** 10.3390/mps4020024

**Published:** 2021-03-30

**Authors:** Rebecca Grant, Karen Coopman, Sandro Silva-Gomes, Jonathan J. Campbell, Bo Kara, Julian Braybrook, Jon Petzing

**Affiliations:** 1 Wolfson School of Mechanical, Electrical and Manufacturing Engineering, Loughborough University, Loughborough, Leicestershire LE11 3TU, UK; j.petzing@lboro.ac.uk; 2 Department of Aeronautical, Automotive, Chemical and Materials Engineering, Loughborough University, Loughborough, Leicestershire LE11 3TU, UK; k.coopman@lboro.ac.uk; 3 GlaxoSmithKline, Gunnels Wood Road, Stevenage SG1 2NY, UK; sandro.gomes@mail.com (S.S.-G.); bo.kara@evoxtherapeutics.com (B.K.); 4 LGC Group, Queen’s Road, Teddington, Middlesex TW11 0LY, UK; jonathan.campbell@LGCGroup.com (J.J.C.); julian.braybrook@LGCGroup.com (J.B.)

**Keywords:** measurement uncertainty, Flow Cytometry, subjectivity, variation, data analysis

## Abstract

Measured variability of product within Cell and Gene Therapy (CGT) manufacturing arises from numerous sources across pre-analytical to post-analytical phases of testing. Operators are a function of the manufacturing process and are an important source of variability as a result of personal differences impacted by numerous factors. This research uses measurement uncertainty in comparison to Coefficient of Variation to quantify variation of participants when they complete Flow Cytometry data analysis through a 5-step gating sequence. Two study stages captured participants applying gates using their own judgement, and then following a diagrammatical protocol, respectively. Measurement uncertainty was quantified for each participant (and analysis phase) by following Guide to the Expression of Uncertainty in Measurement protocols, combining their standard deviations in quadrature from each gating step in the respective protocols. When participants followed a diagrammatical protocol, variation between participants reduced by 57%, increasing confidence in a more uniform reported cell count percentage. Measurement uncertainty provided greater resolution to the analysis processes, identifying that most variability contributed in the Flow Cytometry gating process is from the very first gate, where isolating target cells from dead or dying cells is required. This work has demonstrated the potential for greater usage of measurement uncertainty within CGT manufacturing scenarios, due to the resolution it provides for root cause analysis and continuous improvement.

## 1. Introduction

Variability within Cell and Gene Therapy (CGT) manufacturing arises from numerous sources and it has been well documented that starting material [1,2], inter-site comparability [3] and final product comparability [4] to name a few examples cause variability. However, operators are a key function of the manufacturing processes and cause inherent variation due to numerous factors, such as experience with a specific instrument, assay or specific cell lines [5]. Other manufacturing and safety critical industries have reported the influence of the operator within “normal” and stressful scenarios, as well as decision-making and treatment options in medical scenarios [6,7,8,9,10]. Medical error has recently been estimated to be the third biggest killer in the US, following cancer and heart disease, if it were to be listed as an International Classification of Disease code [11]. This is described as a function of human and system factors, communication breakdowns, diagnostic errors, poor judgement and inadequate skill within medical professionals. Human error is often mentioned as a cause of variation; however, safety and reliability research often refutes this, stating it is not the human themselves, but an understanding of the whole system [12].

Flow Cytometry (FC) as a core platform for CGT measurement has been the subject of beneficial standardisation efforts within the many elements of this measurement process [13,14,15]. For example, Optimized Multicolor Immunofluorescence Panel (OMIP) reference procedures provide operators with well-established assays to follow to reduce variation [15]. The bulk of this standardisation work occurs in the pre-analytical and analytical phases, covering sample preparation, equipment setup and running the assay. In addition, there are opportunities to take part in External Quality Assessment (EQA) [16] schemes such as those run by UK-NEQAS or equivalent. However, little research exists to quantify the impact of post-analytical Flow Cytometry, focusing specifically on the data analysis (gating) procedure, commonly performed by subjective manual population segmentation analysis. Our recent study has shown the variation of multiple operators analysing the same Flow Cytometry data across a 3-step gating procedure, showing the variability that opinion and equipment optimisation can have on downstream analysis and variability [5].

There has been significant progress in automated data analysis in recent years with multiple efforts in developing supervised (and non-supervised) machine learning algorithms to analyse FC data through cluster analysis and pattern recognition [17,18,19,20]. Whilst these provide significant and varied toolsets for analysing highly dimensional data in a shorter time (reducing human input and human factored measurement variation), the potential for intrinsic variability of these methods results in the need for an independent evaluation through a validation process. Importantly, the interface between operators and algorithms may likewise need to be better understood, to reduce variability and increase the quality of data passed on for manufacturing and treatment decisions. This interface has been shown to be of increasing importance in technologies relying on subjective analysis (i.e., histology [21]).

Process variation is already measured in the context of the Coefficient of Variation (CV)—a common measure of variability in assays and outcomes. Whilst CV is a heavily utilised statistic for reporting variation within healthcare literature, it typically only represents end-point variation, and it is difficult to use CV to provide localised information within (for instance) a gating sequence [22]. Repeatability and reproducibility go hand in hand, but further traceability and understanding of where variability can impact the product and manufacturing process are necessary for improved control and efficient therapeutic delivery [4,23]. Within traditional manufacturing scenarios, measurement uncertainty is an already well utilised method of calculating variability contributed to from a number of sources into a measurement [24,25,26,27], typically following the Guide to the Expression of Uncertainty in Measurement (GUM) for source identification and calculation [28]. Building up this uncertainty budget allows for greater resolution into individual factors affecting variation, and enables further investigation when uncertainty appears to be out of specification. In addition, uncertainty can then be linked to manufacturing Internal Process Control (IPC) and methods allowing clear visualisation of methodology and a better identification of where variances can arise. Based upon previous work from our group, an understanding of uncertainty is needed by those who can make influential decisions based upon the measurement reports they receive, to be more confident in their judgement of a situation [29].

Previous work has explored the potential for manual gating operator variation within a simple embryonal carcinoma 2102EP cell model with 3 gating stages [30]. However, whilst this study initially explored uncertainty measures as a potential for use, the minimal gating stages did not present enough complexity of analysis to fully extol the potential of uncertainty analysis in comparison to CV for the identification of high variance or problematic gates within a sequence. Consequently, this new research explores and demonstrates increasing operator variation as a function of increased gating stages measured via systematically applied uncertainty principles as a function of increasing complexity, using a cell analysis exemplar for identifying naïve T-cells from Peripheral Blood Mononuclear Cells (PBMCs). Furthermore, this work demonstrates how measurement uncertainty attributed to post-analytical Flow Cytometry processes can identify problematic gating stages, and the results can be uniquely encapsulated in process control metrics, providing clear decision-making information for process control and underlying operator training needs specifically within the context of CGT. The authors do also acknowledge that all Flow Cytometry assays are unique and varied to suit the appropriate target for the analysis in question, so this research presents a qualitative analysis method to further consider variation in post-processing sequences where appropriate. Gating stages are not strictly independent of each other, but this method provides an opportunity to increase measurement resolution, for greater certainty in the results presented.

## 2. Materials and Methods

The methodology for outlining the file generation and participant study design is detailed here. The naïve T-cell subset was selected for the basis of this research because this is the starting material for current T-cell therapies and provides increased complexity of the data from previous research [30]. Primary Peripheral Blood Mononuclear Cells (PBMCs) were acquired from LGC-ATCC cell banks for use within this phase of research (Cat Number: PCS-800-011, Lot number: 80628171). Confirmation of donor consent from the supplier of this material for research purposes was obtained for this experimentation.

### 2.1. Cell Selection and Culture

A vial of LGC-ATCC PBMCs was thawed and resuspended in RPMI 1640 cell culture media (Cat Number 11875093, Lot Number: 1906058) (fortified with 10% *v*/*v* Fetal Bovine Serum (FBS)). Prior to centrifugation at 300× *g* for 5 min, a sample was taken for an initial cell count. Three counts were completed using a Nucleo-Counter^®^ NC-3000™ and ChemoMetec Via1-Cassettes™, to stain and measure cells with Acridine Orange and DAPI dyes. Once centrifuged, the cell pellet was resuspended in RPMI 1640 fortified media and transferred to a T75 flask at 0.67 × 10^6^ cells/mL. This T75 flask was moved into a humidified incubator at 37 °C with 5% CO_2_, for 24 h to allow the cells to recover from cryopreservation.

### 2.2. fcs File Generation

A series of fcs files were generated using the primary PBMCs kept in culture for the last 24 h.

A sample of cells was counted as described in Section 2.1 and the remaining cell suspension was centrifuged (5 min at 300× *g*) and cells washed twice in Cell Staining buffer before being finally resuspended in 1 × 10^7^ cells/mL. Aliquots 0.1 mL (approximately 1 × 10^6^ cells) were used for all samples: three fully stained samples, one unstained sample, one live/dead stained sample, five single stain controls, five isotype controls, five Fluorescence Minus One (FMO) controls). All samples except the unstained control were stained with 1 μL of BioLegend Zombie Aqua Viability dye (Cat Number: 423101, Lot Number: B243783) for 20 min in the dark. Post-incubation, cells were washed twice with 1 mL Cell Staining Buffer, centrifuged (300× *g* for 5 min) and cells resuspended in 100 μL Cell Staining Buffer. This process was repeated, with all samples aside from isotype controls stained with 5 μL of BioLegend Human TruStain FcX™ Fc blocker (Cat Number: 422301, Lot Number: B235079).

The remaining cells were then stained according to the manufacturer stain protocols adding 5 μL of respective stain (Table 1) to the samples and controls. All samples and controls were incubated in the dark at 4 °C for 30 min. Once incubated, the cells were washed twice and transferred to BD Falcon™ Round Bottom 12 × 75 mm tubes (Cat 352063) and kept covered to minimise light exposure. Cells were run through a BD FACSCanto™ II Flow Cytometer, using the respective fluorescence channel and voltage: FSC 310 V, SSC 400 V, FITC 389 V, APC 420 V, APC/Cy7 472 V, PE 350 V, BV421 300 V and BV510 451 V for the live-dead stain, once a daily calibration was completed using CS&T beads (Lot: 74538, Successful calibration).

Each tube and respective fcs file were generated using a medium flow rate (60 μL/min) and by acquiring 30,000 cellular events. Three stained sample fcs files were generated to build a library of repeats to use within the variation studies, alongside the control files listed. Files were exported as fcs 3.0 version types for use in Flowjo Version 10.0.8r1 third party analysis software [31] and saved as a workspace.

### 2.3. Flow Cytometry Study Organisation

A total of 23 participants from three separate centres (5 from an academic institution, 13 and 5 participants respectively from two separate industrial companies) were individually invited to complete the study in a quiet analysis space. Participants had a range of experience (in terms of length of time) using Flow Cytometry, from 1 month through to over 10 years. There was also variation in how frequently participants used Flow Cytometry, from multiple times per week to a few times each year.

Study sessions had a one-hour maximum duration, and participants were shown three FlowJo workspaces, which contained three fully stained PBMC.fcs files. FlowJo was the choice of analysis platform due to access of the software across all three collaborator and participant sites, meaning a higher number of participants were likely to be familiar with the platform. Identical files were included in each workspace (Phase 1), and participants were instructed to use their experience and gate through a five-plot sequence to identify target cells (using Forward Scatter (FSC) plot against Side Scatter (SSC)), single cells, live cells, CD3+ cells and finally to apply a quadrant gate to the double positive naïve T-cell CD4+ CD45RA+ population to identify final positive population cell counts. The CD4+ CD45RA+ pipeline was kept, because of the suitability of this panel to current engineered T-cell product panels and to provide a good basis to increase complexity for the subsequent studies to more representative CGT T-cell product analysis. Participants were informed that these files were in fact repeat measures taken at one time in a Flow Cytometry Assay, so they would be aware of their similarity, but not necessarily identify that files were identical to reduce any “copy across” behaviour. Files were labelled according to this, so they appeared as experimental repeats, rather than identical files.

**Figure 1:**
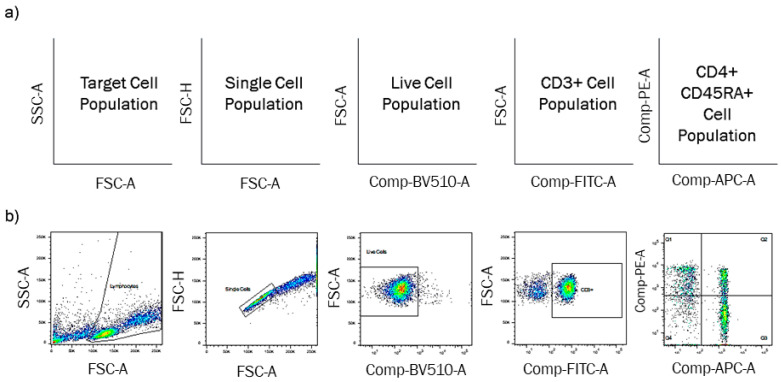
(**a**) Gating sequence participants were asked to follow, to identify the target single live cell population, with CD3+ CD4+ CD45RA+ for naïve T-cells. (**b**) Second gating sequence participants followed (Phase 2), identical to (**a**) but with additional diagrammatical information of the plots and gates, which participants had to copy.

Participants were also provided with isotype controls and FMO controls in each workspace to aid gate application and were allowed to freely select the manual gating tool of their choice within FlowJo. The gating sequence participants were asked to follow is shown in Figure 1a, and participants gated each workspace of files separately to ensure a correct quantification of uncertainty through standard deviation calculation described in the following section.

Participants took part in a second phase (Phase 2), where they repeated the gating process for the CD3 + CD4 + CD45RA + cell population but were asked to follow a diagrammatical protocol to apply gates instead of using their own judgement, shown in Figure 1b. This protocol identified the gate shape and position for each of the five stages, as defined by the research team. To remove additional variability when placing these gates, participants were only given the three fully stained samples to apply the gates in each workspace, so no control files could influence gate placement once the images had been copied, and participants followed the same gating sequence provided in Figure 1b. All participants and their respective data (Phases 1 and 2) were anonymised at the point of data collection, and data were stored in accordance with the ethical clearance obtained.

### 2.4. Uncertainty Calculation

**Figure 2:**
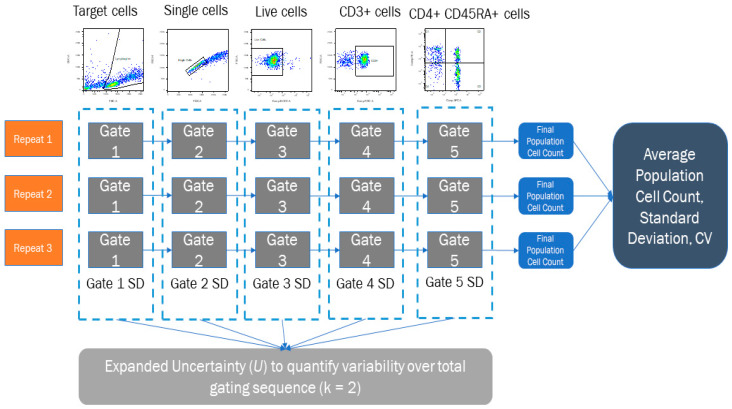
Processing sequence for participant studies to calculate average cell counts, SDs and CVs across three repeats, as well as the Expanded Uncertainty from SD of each of the individual gate repeats (dashed line).

Once the gating studies had been completed, target cell, single cell, live cell, CD3+ and CD4+ CD45RA+ cell population metrics were extracted from the data, using the results from the identical repeated file situated in each FlowJo workspace. These were then used to calculate a mean cell count, Standard Deviation (SD) and Coefficient of Variation (CV) for each full gating sequence, per participant using Microsoft Excel software (Office 16), shown in Figure 2. A Combined Uncertainty (*u_c_*) Equation (1) was calculated using GUM principles by combining these Type A uncertainties by summation in quadrature [28] noting here that Type A uncertainties are measured standard deviations from repeats of experimental data, whereas Type B uncertainties are derived sources other than the experimental data (calibration reports, data sheets, technical specifications etc.) The values calculated within this process do have an interrelation as the subsequent gating steps can depend upon earlier gating analysis and other factors such as markers chosen. However, it should be noted that the methodology within this analysis creates three repeat phases to quantify SD, to consider a basic uncertainty budget around the analyst, rather than the data specifically. Uncertainty budgets can be built to whatever resolution is required and tailored to specific analytical scenarios. This research presents one novel way of using uncertainty quantification for analyst post-processing, to get an understanding of Human Resource variance in Flow Cytometry analysis. The decision of what should be factored into the final uncertainty budget should always be reviewed, to consider the most relevant contributions, of which the analyst should have a self-awareness.

A full explanation of the measurement uncertainty calculation process, along with an example calculation from this experiment are detailed in the Supplementary Material. This includes the combination of gating steps described, as well as other considerations that can be included if the uncertainty budget is expanded to include other sources of variation. Whilst uncertainty may be very small when contributing to a larger uncertainty budget, this method allows that resolution to be identified for the analyst to consider appropriate features for inclusion/exclusion.

The *u_c_* value was expanded with a coverage factor of *k* = 2 Equation (2), representing a +/− 95% Confidence Interval for the uncertainty statement, which gave each participant a representative Expanded Uncertainty figure (*U*), to show individual variance.

(1) $$u_{c}=\sqrt{{\mathrm{SD}}_{\begin{array}{c} \mathrm{Target}\\ \mathrm{Gate}\;1 \end{array}}^{2}+{\;\mathrm{SD}}_{\begin{array}{c} \mathrm{Single}\\ \mathrm{Gate}\;2 \end{array}}^{2}+{\mathrm{SD}}_{\begin{array}{c} \mathrm{Live}\\ \mathrm{Gate}\;3 \end{array}}^{2}+{\mathrm{SD}}_{\begin{array}{c} \mathrm{CD}3+\\ \mathrm{GATE}\;4 \end{array}}^{2}+{\mathrm{SD}}_{\begin{array}{c} \mathrm{CD}4+\mathrm{CD}45\mathrm{RA}+\\ \mathrm{Gate}\;5 \end{array}}^{2}}$$

(2) $$U=k\;\times \;u_{c}$$

## 3. Results

Twenty three participants took part in the Phase 1 study (analysis by personal judgement whilst 20 of the 23 initial participants took part in the Phase 2 study (analysis following a diagrammatical protocol). This set of studies aimed to quantify an initial operator uncertainty, where a desirable uncertainty was initially unknown. Therefore, statistical power and ideal sample sizes could not be quantified before the experiment, they could only be quantified Post Hoc, for potential validation purposes.

Using G*Power software [32], the power calculations indicated that the participant cohort was sufficient to meet any statistical differences attributed to the conditions under a test (at a significance level (p) of 0.05 to a minimum power of 0.80 for percentage cell count results (comparison of mean cell counts).

**Figure 3:**
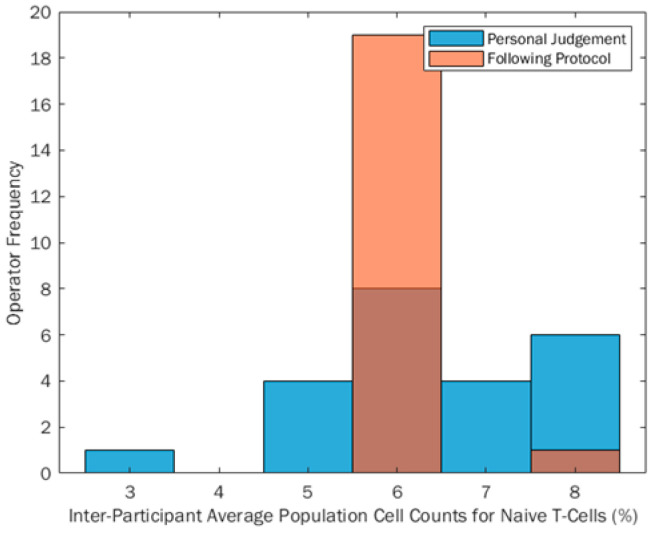
Histograms showing percentage cell counts (shown as a percentage as a function of total cells in the sample) reported when participants gate using their own judgement (blue bars) and when following a protocol (orange bars).

The inter-person range of % positive CD3 + CD4 + CD45RA + cell counts reported from Phase 1 to Phase 2 reduced by 57%, indicating that when participants follow prescribed gating protocols this can reduce the reported range of percentage cell counts. This shows an improvement in reproducibility of Flow Cytometry data, by quantifying the variation input to the final measurement from the participant gating the post-analytical data alone. This can be seen in Figure 3, with a much smaller percentage range when participants gated following the protocol (orange bars) compared to gating using their own judgement (blue bars). Any darker areas shown are an overlap of the two distributions. It can also be seen through descriptive statistical tests that the percentage cell count data became more skewed when analysing following a protocol (skewness *z*-score 5.68), from a flat, yet normal distribution when analysing data using own judgement (skewness *z*-score −0.73). IBM SPSS Statistics Version 24 was used to calculate descriptive statistics metrics and skewed data is defined by skewness *z*-scores being outside a ±2.58 boundary. Anything within this boundary is therefore assumed to be normally distributed. When comparing this to the visual plot in Figure 3, the data appear skewed due to the uniformity of the inter-participant percentage cell counts, which will impact the skew calculation.

A Sign statistical test was also conducted to compare the equality of the medians of the two groups and it was found that there was no significant difference between the absolute cell count medians of the two test conditions (*p* = 0.824). This suggests that protocols are beneficial for gating because the location metric for the absolute cell count is unchanged. The main location value remains unchanged, but using a protocol has been shown to reduce variability around this location metric (median), inferring that we can potentially achieve more uniform inter-participant results using diagrammatical protocols, without showing a shift in overall reported values.

**Figure 4:**
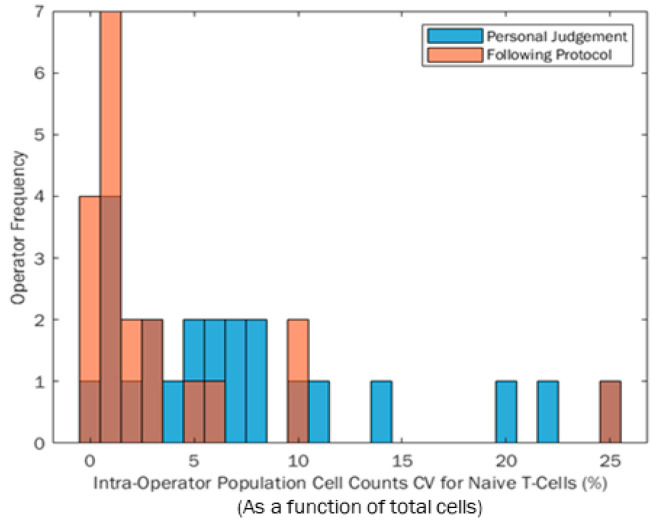
Histogram of participant CVs when repeat gating has the same data using their own judgement (blue bars) and then following a protocol (orange bars).

The CV for each participant was quantified from the three repeats conducted within each Phases 1 and 2 session, which can be seen for the participant population in Figure 4. The blue distribution indicates participant CVs from their own judgement in Phase 1. The orange distribution relates to the CVs quantified when participants followed the same protocol to analyse the data. Any dark areas are an overlap of the two histograms. The range of both distributions are the same (25% range in percentage cell count); however, the population became more positively skewed when participants followed the protocol. In this instance, a positive skew towards zero is likely to be preferable, because more participants have a lower final CV which is preferable for reproducibility of results. Aside from the one participant with the largest CV when following the protocol in Figure 4, using a protocol appears to reduce the range of participant CV, making data more repeatable between participants.

**Figure 5:**
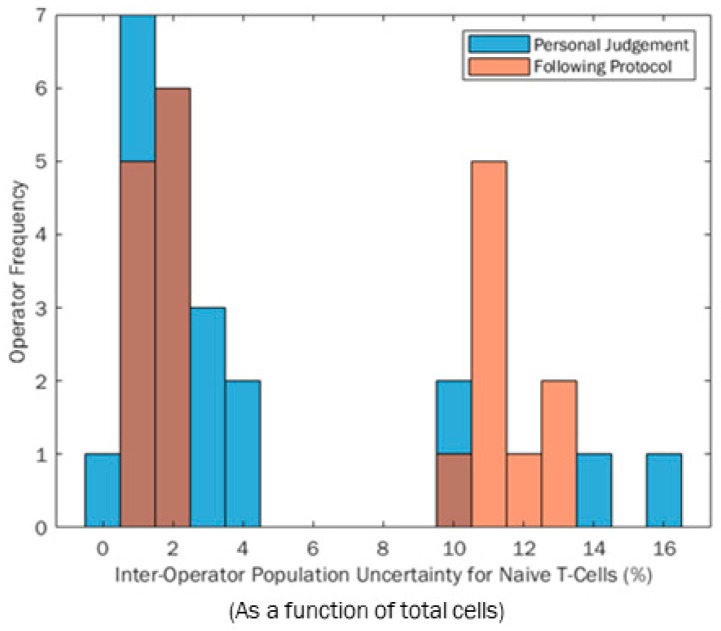
Comparison histograms of participant expanded uncertainties (U) when gating using their own judgement (blue bars) and when following a protocol (orange bars).

Participant measurement uncertainty was calculated by combining the SDs from each gate in quadrature as described in the methodology Equation (1). Figure 5 shows the distributions of these participant expanded uncertainties (U) when gating based on their own judgement (blue) and following the protocol (orange). Darker areas on the histogram are overlaps. The range of measurement uncertainties is smaller when participants follow the protocol (not correlated to CV); however, an unexpected bimodal distribution can be seen for both histograms. Similarly, it can be seen that the bimodal peaks are more refined when participants use a protocol rather than their own judgement. This infers that using a protocol could better influence measurement reproducibility between operators because it focuses on the majority of participants around a central point, similar to a standard metric, although it is more likely to be influenced by extreme values. However, a bimodal distribution was unexpected in both test scenarios. This was due to participants completing repeats, where some repeats were very similar in location, giving a low variance cluster. Other participants had repeats that included significantly more or less cells in certain repetitions, causing a rise in total variance, as shown by the “high variance” cluster of results. A deeper investigation shows that those who were more variable in repetitions were impacted by boundary effects on the edge of the Flow Cytometry plot in the first gating step. Any cell scatter data gathered that did not fall in the bounds of the axes was appended to the outer axes of the scatter plot. If the participant is not aware of this feature and gates to or off the edges in a particular step, they can include and exclude these data. The irregular inclusion and exclusion of these data across three repeats is what caused the bimodality within this research. The lower cluster repeatably includes or excludes this dataset, whilst the higher group mix across the repeats, causing greater variability.

**Figure 6:**
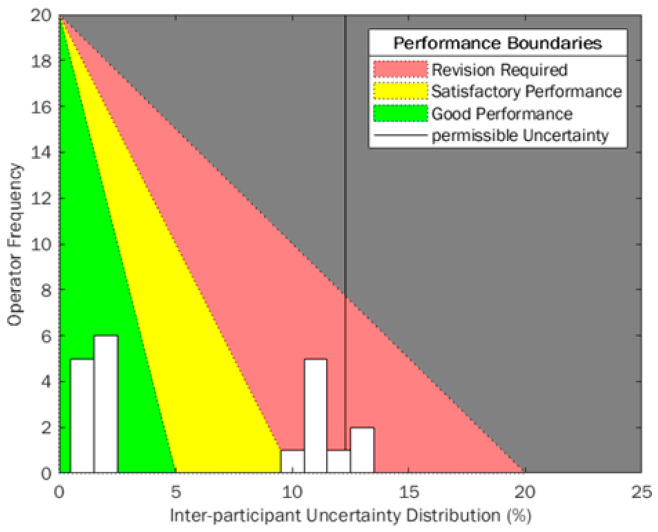
Uncertainty histogram from participants using a protocol applied to a potential traffic-light quality monitoring system.

To better monitor the uncertainties calculated for each participant, an example histogram (expanded uncertainty calculated when participants followed a protocol, (Phase 2)) has been overlaid on a traffic-light style quality performance diagram similar to that used in other industrial sectors (Figure 6). Uncertainty is built up from smaller uncertainty measurements that are combined according to Equations (1) and (2), so it is an important metric to utilise in variation-monitoring scenarios because root case analysis can be investigated more thoroughly, reliably and quickly. Using uncertainty means the SD of each gate applied in the sequence must be calculated, giving a better resolution to the uncertainty when results outside of acceptance regions occur. CV provides a variation metric at the final point in the process, which can be challenging to break down into smaller contributing components when out-of-boundary measurements are obtained. However, because of its thorough use in experimental reporting, a consideration of CV and uncertainty together provide a great resolution and understanding of the process and could qualitatively enhance process control and improvements over time.

This diagram uses a green-amber-red system (RAG) to align to pre-defined acceptance criteria for variability within a manufacturing scenario. Traditionally, these color acceptance boundaries would be vertical stripes to identify the separation between sections. The RAG traffic light system is very identifiable across many different cultures and industrial sectors but can have different meanings between the three phases. Red is always identified with “stop” or “bad”, and green with “go” or “good” respectively. It has been identified that RAG systems function best when all three states are well defined, and all users understand the boundaries and movement between states [33]. In this instance, an ideal variation would be zero, therefore having a strong positive skew, so an equation of a straight line has been used to adjust these boundaries. The number of total participants or employees in a laboratory or facility is set as the *y*-intercept, because ideally everyone would have low variance, as close to zero as possible, so this is the ideal. *X*-axis boundaries are defined by acceptance criteria for “good”, “satisfactory” and “revision required” regions. In this instance, these values have been adjusted as a function of the International Council for Standardisation in Haematology (ICSH) and the International Clinical Cytometry Society (ICCS) acceptance criteria for imprecision, where satisfactory CV is <10% [34]. This was halved to identify “good” performance and was doubled to identify where “revision required”. Currently, no acceptance criteria for uncertainty of personnel exists, so these values were assumed from the CV exemplar in this instance, but can be adjusted to suit assay application and internal metrics.

Using this triangle-oriented diagram in Figure 6, there will be some instances where overlaid histogram bars will possibly span across two acceptance boundaries, for example, at 10% uncertainty the histogram bar crosses “satisfactory” and “revision required” regions. Technically, all would fall within “satisfactory”. However, higher participant frequency infers that more training may be required to reduce uncertainty across participants/employees, because others have been able to attain lower uncertainties. This diagram is designed to be a quick indicator for laboratory variability, due to the RAG gradients making it easy to show performance at intervals. The angled boundaries provide a cognitive behavioural change, showing viewers that lower variance and positive skew is desirable when monitoring variance and uncertainty.

Statistical power was also calculated for the two uncertainty distributions, to identify whether there was a suitable sample size for the uncertainty comparison between the two phases. Using the same assumptions for power (ideal = 0.80) has been used in this instance. The actual power achieved through this study with the reported number of participants is 0.213. This low power indicates that any differences seen from the data have a low probability of being just due to the two test conditions used and no other underlying factors present. This needs to be considered carefully, because a greater number of participants could always benefit and provide greater confidence in results; however, in this instance the distributions are not normal. The variance calculated for power assumes a distribution with central tendency.

## 4. Discussion

Within CGT products, cell counts are a key metric reported back across a variety of clinical and manufacturing tests. Cell count is influenced by variation in upstream biological processing and downstream manufacturing processing; however, a key facet that has not been fully explored is the potential for metrological variation being introduced by human operators performing manual gating of low and high dimensional flow cytometry data. This has been explored through a comparative study using five stage gating process of PBMC files, comparing operator native performance versus performance driven by protocols. In addition, this work has concentrated on defining the benefits of measurement uncertainty techniques for identifying problematic variation within the processing (between gated steps) in alignment with more traditional CV-based final gating variations, and the introduction of process monitoring tools.

CV is a common reporting metric for variability. CV is generally calculated from repeat measures of the final count, and therefore (typically) there is no further resolution to the CV-based variation analysis to help identify different individual sources of variation (e.g., at each gating stage). The range in operator native performance CV reported from the author’s previous published work looking at a simplified three gate sequence was 6% between participants [30], whereas in this five-gate research the between-participant CV range increased to 22%. This further acknowledges that as post-processing Flow Cytometry data sequences become more complex and the variation attributed to a person completing the analysis increases significantly. Whilst the range of CV between study participants did not reduce when participants followed protocols (because of one poorly performing participant), the CV became more positively skewed towards zero. This is desirable for CV (and other variance metrics) to reduce them as it limits the inherent variability from the participant/analyst into the final measure.

Measurement uncertainty has previously been shown to be an effective way of monitoring human factor variability through structured repeat measures. It provides an additional layer of specificity to the variance analysis to identify specific steps in the measurement process that can then become the focus of continuous improvement. Measurement uncertainty was determined by calculating individual standard deviations from each gate and then combining in quadrature. This process gave more resolution to identify specific sources of variation that contribute to a participant’s overall variability. Expanded measurement uncertainty, U (expressed at a coverage factor of *k* = 2 or 95% confidence limits), increased from 12% (from an initial, simplified 3-step- previously published by our group) to 16% the range between-participants when following this current 5-step procedure respectively. This comparison of participants was using equivalent Phase 1 operator native behaviour, where they followed an axis setup and applied their own gates, in comparison to physically copying gates in a defined protocol during Phase 2.

Overall, participants following a diagrammatical protocol-reduced variation in absolute cell counts reported and also reduced participant uncertainty from repeated measures. Inter-participant variability was reduced by 57% when study participants followed a protocol. A statistical Sign test also showed no significant difference between reported absolute cell counts between Phases 1 and 2, showing that protocols can produce similar results to the null testing condition. In addition, the range of expanded measurement uncertainty (U) was also reduced (by 23%) when participants followed the protocol, and using this method also allowed elements of the protocol that caused the inherent variation to be identified.

Median expanded uncertainty had no significant change between Phases 1 and 2 (2.1% and 2.2% respectively), indicating that a good proportion of participants were not individually variable through the study. The median is a more reliable statistic in this instance due to the separation of the data into two clear groups. Mean uncertainty for Phases 1 and 2, respectively, was 3.8% and 5.8%. This demonstrates the importance of visualising data and not just relying on “normal” statistical measures, which is especially clear in Phase 2, where the mean uncertainty of the groups is not located near any peak maxima. High values in uncertainty results were generally identified as being due to participant variability in applying the first gate to separate target cells from dead or dying cells. In addition, there was extra variation here due to boundary effects on the far edge of the plot, caused by data that would otherwise be outside the plot axes.

This research has translated and applied visual toolsets from other manufacturing sectors with a RAG system to give rapid indications of current performance metrics and has been shown to be applicable to the detailed uncertainty analysis as well as CV-based analysis. This process monitoring tool shows angled acceptance regions and limits rather than set bandwidths as an initial move to identify variability through cognitive indicators. This is an example of how a monitoring system can be used for continuous improvement of variable factors, and illustrates a novel, clear reporting system for CGT cell measurement variability, to highlight the importance of monitoring variation as well as measurements of location such as mean and median. Variability should always aim to be low for increased reliability and confidence, so the adjustment in process monitoring tool limit angle provides an intuitive indication for all to see real-time performance and drive the investigation of root causes of higher variability.

Human variability is a key source of variance that must be taken into sensible consideration. Measurement uncertainty does take more time to measure and analyse, but the output value provides much greater resolution of understanding and confidence in reporting. The ability to identify variance from contributing gate steps showed the first stage to be the most variable for participants, but it is important to highlight that often the gating stages are interconnected, and so a full review of the process should take place when monitoring in this detail. However, because (in reality) measurement uncertainty is built from multiple sources, other variation factors should be considered within future uncertainty budgets. Whilst in this research, gating sequence steps were combined to define operator variance, other additional upstream and downstream sources may need to be considered. For example, environmental effects, sample preparation procedures, Flow Cytometer uncertainty and instrument calibration all contribute to variability with a knock-on effect to the final measurand reported. In addition, whilst the potential for using protocols offers reduction of variability, it is important to identify that following a protocol is challenging. It increases time taken for analysis, and over the course of time operators will commit information to memory, developing tacit knowledge, and a physical protocol will not be used as often as intended.

Identifying the effectiveness of applying measurement uncertainty principles (in addition to traditional CV measures) to understand operator variation, and the use of process monitoring tools linked to these principles has been demonstrated. This enhanced level of information potentially enables continuous improvement and enhanced decision making in a way that CV may not be able to support in isolation. Within a CGT context, this poses a benefit wherever measurement is applied, from initial starting material quantification to final release of the CGT product. Considering the operator more thoroughly through this process will help to improve inherent quality for all.

## Figures and Tables

**Table 1:** Antigen markers and respective isotype controls.

Antigen Markers	Respective Isotype Control
BioLegend FITC anti-human CD3 antibody (Cat Number: 300306, Lot Number: B218086)	BioLegend FITC Mouse IgG2a ĸ Isotype Control antibody (Cat Number: 400207, Lot Number: B235551)
BioLegend APC anti-human CD4 antibody (Cat Number: 357405, Lot Number: B223335)	BioLegend APC Mouse IgG2b ĸ Isotype Control antibody (Cat Number: 400329)
APC/Cy7 anti-human CD8 antibody (Cat Number: 300926, Lot Number: B231191)	APC/Cy7 Mouse IgG1 ĸ Isotype Control antibody (Cat Number: 400127, Lot Number: B235070)
BioLegend PE anti-human CD45RA antibody (Cat Number: 362552, Lot Number: B210221)	PE Mouse IgG2b ĸ Isotype Control antibody (Cat Number: 400313, Lot Number: B246304)
BioLegend BV421 anti-human CD56 antibody (Cat Number: 423101, Lot Number: B246952)	Brilliant Violet 421 Mouse IgG1 ĸ Isotype Control antibody (Cat Number: 400157, Lot Number: B237449)

## Data Availability

Data are not available for distribution from this study.

## Supplementary Materials

The following are available online at https://www.mdpi.com/article/10.3390/mps4020024/s1, Figure S1, Process flow of calculating measurement uncertainty, with a Flow Cytometry gating exemplar provided for illustration in this context.
