# Supplementary material for: Assessment of Protocol Impact on Subjectivity Uncertainty When Analyzing Peripheral Blood Mononuclear Cell Flow Cytometry Data Files
Source: Methods Protoc. 2021 Mar 30;4(2):24. doi: 10.3390/mps4020024 (PMC8103269; doi:10.3390/mps4020024)
Supplement: Supplementary file 1 [file mps-04-00024-s001.pdf]

The following diagram workflow shows how to calculate measurement uncertainty, as detailed in the Guide to Expression of Uncertainty in Measurement [1] [28], with an example of how uncertainty was calculated within this research paper. This shows how different sources can be identified and combined to create a more confidence expression of uncertainty of a measurement.

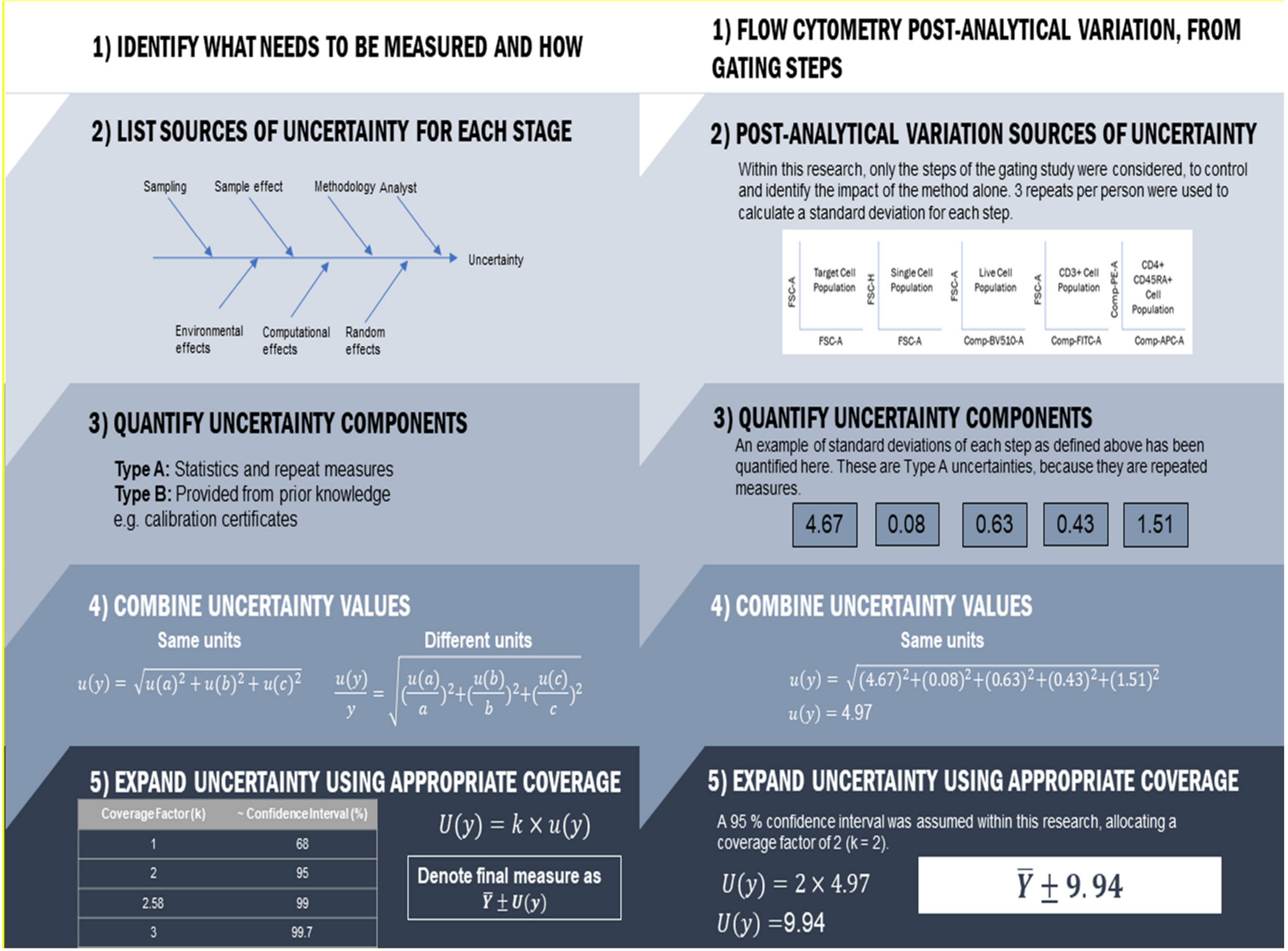

Figure S1. Process flow of calculating measurement uncertainty, with a Flow Cytometry gating exemplar provided for illustration in this context.

Reference

1. Lequin, R.M. Guide to the expression of uncertainty of measurement: Point/counterpoint. *Clin. Chem.* **2004**, *50*, 977–978, doi:10.1373/clinchem.2003.030528.
